# Epidemiology, species distribution, and predictive factors for mortality of candidemia in adult surgical patients

**DOI:** 10.1186/s12879-020-05238-6

**Published:** 2020-07-13

**Authors:** Wei Zhang, Xingpeng Song, Hao Wu, Rui Zheng

**Affiliations:** 1 Department of Pulmonary and Critical Care Medicine, Shengjing Hospital of China Medical University, No. 36 Sanhao Street, Heping District, Shenyang, 110004 Liaoning China; 2 Department of Pulmonary and Critical Care Medicine, Anshan Central Hospital, No. 77 Nanzhonghua Street, Tiedong District, Anshan, 114000 Liaoning China

**Keywords:** Candidemia, Surgery, *Candida parapsilosis*, Risk factor, Mortality

## Abstract

**Background:**

We evaluated the epidemiology, clinical characteristics, microbiology, outcomes, and risk factors for mortality of candidemia in adult surgical patients in Shenyang from 2012 to 2018.

**Methods:**

We designed a retrospective observational study of adult patients with candidemia in a teaching hospital including three hospital campuses. Data regarding clinical and demographic characteristics were collected from the patient’s medical records.

**Results:**

Of the 236 cases of candidemia, 172 (72.9%) were identified in surgical patients, including 146 (84.9%) general surgeries, 11 (6.4%) urologic surgeries, 6 (3.5%) thoracic surgeries, and others. Higher proportions of solid tumors, total parenteral nutrition, the presence of a urinary catheter, and the presence of a gastric tube were observed in surgical patients with candidemia versus non-surgical ones, whereas the percentages of hematological malignancy, diabetes mellitus, and renal replacement therapy were relatively lower in surgical patients. Renal failure, leukopenia, and thrombocytopenia were less common laboratory findings in surgical patients with candidemia than compared to non-surgical ones. Among surgical patients with candidemia, *Candida parapsilosis* was the predominant species (43%), followed by *C. albicans* (33.7%), *C. glabrata* (11%), *C. tropicalis* (8.1%), and others (4.1%). Overall susceptibility, susceptible dose dependent or intermediate susceptibility, and resistance to fluconazole were detected in 73.3, 19.8, and 3.5% *Candida* isolates from surgical patients, respectively, but no resistance to amphotericin B was observed. Overall, the 30-day mortality in surgical patients was 19.2%. At multivariable analysis, independent risk factors for death in surgical patients with candidemia were ICU stay, thrombocytopenia, and *C. albicans* infection.

**Conclusions:**

Surgical patients account for the majority of candidemia cases. Among patients with recent surgery, risk factors for species distribution, antifungal sensitivity patterns of *Candida* isolates causing candidemia, and independent risk factors for mortality should be evaluated and considered for a better outcome in the antifungal treatment.

## Background

Several studies have reported that *Candida* species account for up to 9–22% of all nosocomial bloodstream infections (BSIs) [1, 2], which implies high-mortality rates and prolonged hospitalization, as well as increased hospital costs [1, 3–5]. Prior surgery has been demonstrated to one of the main risk factors of candidemia [6, 7]. Over the past decades, the proportion of surgical patients in candidemia cases has shown an increasing trend [8]. In recent years, it is reported that approximately 50–55% of patients with candidemia have undergone recent surgery prior to the development of candidemia [4, 5, 9]. The risk of occurrence of candidemia among patients who underwent a surgical procedure or had a central venous catheter (CVC) were 11 times higher than those who did not have a surgical procedure or a CVC [6]. The previous prospective multicenter study conducted in surgical intensive care units (ICUs) has identified several risk factors independently associated with the increased risk of candidemia, including prior surgery, acute renal failure, receipt of parenteral nutrition, and, for postsurgical patients, presence of a triple lumen catheter [6].

Some studies on candidemia have been performed in surgical and critically ill patients [6, 10] or surgery wards [11], however those focusing on candidemia in surgical patients are limited [12]. Therefore, this retrospective study was undertaken to evaluate the epidemiology, clinical and microbiologic characteristics, outcomes, and prognostic factors of candidemia in adult surgical patients in Shenyang.

## Patients and methods

### Subjects and data collection

A retrospective observational study on adult (age ≥ 14 years old) hospitalized patients of candidemia was conducted from January 2012 to December 2018 at a tertiary grade A comprehensive hospital located in Shenyang, China. The setting is an over 6000-bed university-affiliated teaching hospital including three hospital campuses.

Candidemia was defined as the isolation of *Candida* spp. from at least one blood culture in patients with clinical signs and symptoms of infection. For patients with multiple positive blood culture, only the first case of candidemia was included, and furthermore a new episode of candidemia was defined if more than 30 days. The onset of candidemia was defined as the date when the first positive blood culture was collected. One patient undergoing subtotal gastrectomy was excluded as the identification of *Staphylococcus epidermidis* and *C. glabrata* from the same blood culture were both considered contaminants. Besides those lost to follow-up within 30 days after the onset of candidaemia were excluded. Surgical patients refer to those undergoing any recent surgery. Recent surgery was defined as within 3 months of surgery from the onset of candidemia, while other predisposing factors, such as ICU stay, total parenteral nutrition (TPN), presence of CVC, mechanical ventilation, presence of urinary catheter, presence of gastric tube, were defined as occurring within 30 days before the onset of candidemia. Prior antibiotic exposure and prior antifungal exposure were defined as the administration of any antibiotics or antifungal drugs during the 30 days before the onset of candidemia. Antifungal therapy was considered as empirical if any antifungal therapy started within 24 h of the time the blood culture specimen was drawn but before the species identification, and as targeted after the species identification. Abdominal surgery refers to any operation involving organs including the stomach, small intestines, the colon or rectum, the gallbladder, the liver, pancreas, the spleen and the appendix. The abnormal laboratory results were defined as follows: renal failure (a creatinine clearance < 60 mL/min), anemia (a hemoglobin level of < 110 g/L in women and < 130 g/L in men), hypoalbuminemia (a serum albumin concentration < 30 g/L), hyponatremia (a serum sodium level < 135 mmol/L), leukopenia (peripheral white blood cell count < 4 × 10^9^ cells/L), and thrombocytopenia (platelet count < 100 × 10^9^ cells/L).

Demographic and clinical data regarding basic characteristics, treatment, and clinical outcomes, laboratory data were collected from the electronic medical records during the study period. The study was approved by the Medical Ethics Committee in Shengjing Hospital of China Medical University (reference number 2019PS636K).

### Microbiological analysis

The microbiological laboratory methods for blood culture, *Candida* species identification, and antifungal susceptibility testing were performed as previously described [13]. The antifungal susceptibilities of the antifungal agents were evaluated according to clinical breakpoints (CBPs) recommended by the Clinical Laboratory Standards Institute (CLSI) [14] or European Committee on Antimicrobial Susceptibility Testing (EUCAST) [15].

### Statistical analysis

Data were analyzed with SPSS (IBM SPSS Statistics 21.0) statistical software. The Kolmogorov-Smirnov test was applied to evaluate the normal distribution of continuous data. Normally distributed continuous variables were expressed as mean ± SEM (standard error of the mean) and compared using Student’s *t*-test. Non-normally distributed data were reported as the median and the 25th–75th percentiles and analyzed with Mann-Whitney *U*-test. Categorical variables were presented as number (%) and compared between groups by the χ^2^ test or χ^2^ continuity correction test. Those variables with a *P*-value < 0.1 in the univariable analysis were entered into the multivariable logistic regression analysis model to determine independent risk factors for 30-day mortality, and the results were presented as odds ratios (OR) with their 95% confidence intervals (CI) and *P*-values. A two-tailed *P*-value of < 0.05 was considered statistically significant.

## Results

**Fig. 1:**
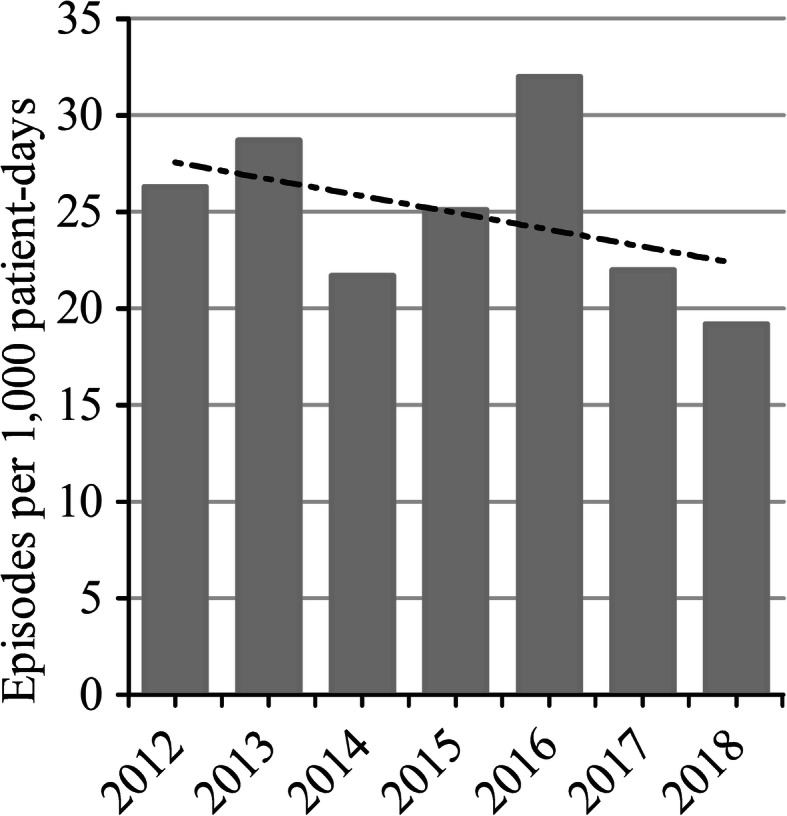
Annual candidemia incidence density in surgical patients, 2012 to 2018

During the study period 2012–2018, we identified a total of 236 episodes of candidemia occurring in 232 adult patients, of whom 172 patients with candidemia had undergone recent surgery with an incidence of 24.8 episodes per 1000 patient-days. Figure 1 presents the annual incidence rates of candidemia in surgical patients using annual patient-days, which varied from 19.2 to 32 episodes per 1000 patient-days during the reported study periods. Overall, the downward trend in the annual incidence rates of candidemia in surgical patients from 2012 to 2018 could not be demonstrated as no statistical significance was found in the linear regression analysis in Fig. 1. (*P* = 0.34).

### *Candida* species distribution

**Fig. 2:**
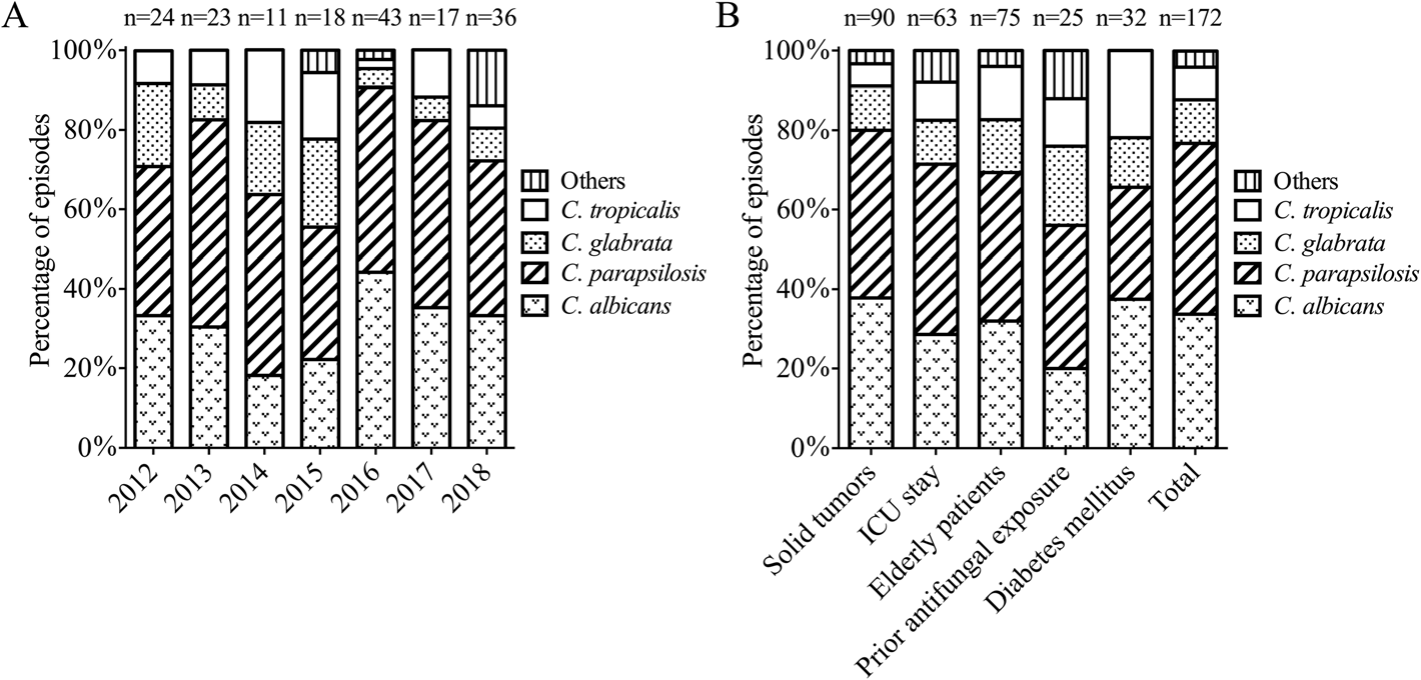
Distribution of *Candida* species isolates from surgical patients with candidemia during the study period according to the time period (A) and underlying diseases or predisposing factors (B)

In the study overall, *C. parapsilosis* (74, 43.0%) was the leading causative organism of candidemia in surgical patients, followed by *C. albicans* (58, 33.7%), *C. glabrata* (19, 11%), *C. tropicalis* (14, 8.1%), *C. famata* (3, 1.7%), *C. guilliermondii* (2, 1.2%), *C. krusei* (1, 0.6%), and one only identified to *Candida* genus level (1, 0.6%). The distribution of *Candida* spp. isolates from surgical patients with candidemia are outlined in Fig. 2. During the 7-year period, *C. parapsilosis* and *C. albicans* remained the first and second most frequently isolated species for each year from 2012 to 2018, respectively. And although *C. glabrata* remained the third most commonly isolated species, its percentage remarkably decreased from 20.8% (2012) to 8.3% (2018) (Fig. 2A). The presence of *C. parapsilosis* in surgical patients was significantly higher than that in non-surgical patients (43.0% vs. 23.4%, *P* = 0.006), while the presence of *C. tropicalis* was lower (8.1% vs. 20.3%, *P* = 0.009) (Table 1). Additionally, among surgical patients with candidemia, *C. tropicalis* isolates were more likely to occur in elderly patients (13.3% vs. 4.1%, *P* = 0.03) and in patients with diabetes mellitus (DM) (21.9% vs. 5%, *P* = 0.005) (Fig. 2B).

**Table 1:** Demographics and clinical characteristics of surgical and non-surgical patients with candidemia

Characteristics	Surgical patients (***n*** = 172)	Non-surgical patients (***n*** = 64)	***P*** value
Age	62.5 ± 14.2	55.0 ± 21.9	0.002
Age ≥ 65 years	75 (43.6%)	22 (34.4%)	0.2
Male sex	119 (69.2%)	41 (64.1%)	0.45
Underlying diseases			
Hematological malignancy	1 (0.6%)	15 (23.4%)	< 0.001
Solid tumors	90 (52.3%)	8 (12.5%)	< 0.001
Diabetes mellitus	32 (18.6%)	24 (37.5%)	0.002
Predisposing factors			
Renal replacement therapy	7 (4.1%)	15 (23.4%)	< 0.001
Current and former smokers	52 (30.2%)	18 (28.1%)	0.75
ICU stay	63 (36.6%)	20 (31.3%)	0.44
Prior antibiotics exposure	166 (96.5%)	52 (81.3%)	< 0.001
Prior antifungal exposure	25 (14.5%)	12 (18.8%)	0.43
TPN	162 (94.2%)	26 (40.6%)	< 0.001
Presence of CVC	67 (39.0%)	26 (40.6%)	0.82
Mechanical ventilation	20 (11.6%)	8 (12.5%)	0.85
Presence of urinary catheter	127 (73.8%)	21 (32.8%)	< 0.001
Presence of gastric tube	132 (76.7%)	23 (35.9%)	< 0.001
Laboratory findings			
Renal failure	31 (18%)	26 (40.6%)	< 0.001
Anemia	144 (83.7%)	55 (85.9%)	0.68
Hypoalbuminemia	71 (41.3%)	31 (48.4%)	0.32
Hyponatremia	73 (42.4%)	18 (28.1%)	0.045
Leukopenia	19 (11%)	19 (29.7%)	0.001
Thrombocytopenia	24 (14.0%)	24 (37.5%)	< 0.001
*C. albicans* infection	58 (33.7%)	25 (39.1%)	0.45
*C. parapsilosis* infection	74 (43.0%)	15 (23.4%)	0.006
*C. glabrata* infection	19 (11%)	10 (15.6%)	0.34
*C. tropicalis* infection	14 (8.1%)	13 (20.3%)	0.009
Fluconazole susceptibility	126 (73.3%)	44 (68.8%)	0.50
Empirical antifungal therapy	115 (66.9%)	40 (62.5%)	0.53
No antifungal therapy	27 (15.7%)	9 (14.1%)	0.76
Outcome			
30-day mortality	33 (19.2%)	18 (28.1%)	0.14
LOS (days)	32 (22.3–47.8)	26 (15–47)	0.02

ICU, Intensive care unit; TPN, Total parenteral nutrition; CVC, central venous catheter; LOS, Length of hospital stay;

### Patient characteristics

One hundred and seventy-two cases had received recent surgery prior to the onset of candidemia, including 146 (84.9%) general surgeries, 11 (6.4%) urologic surgeries, 2 (1.2%) gynecological and obstetric surgeries, 6 (3.5%) thoracic surgeries, 4 (2.3%) neurosurgeries, and 3 (1.7%) orthopedic surgeries. Of these, 119 (69.2%) cases were male, and 33 (19.2%) had CVC-related candidemia. The comparison of demographics and clinical characteristics between surgical and non-surgical patients with candidemia is summarized in Table 1. Of note, surgical patients were older (mean age: 62.5 ± 14.2 years vs. 55 ± 21.9, *P* = 0.002) and had longer length of hospital stay (LOS) (median LOS: 32 vs. 26 days, *P* = 0.02) than those non-surgical patients. In addition, compared with non-surgical patients, the prevalence of solid tumors (52.3% vs. 12.5%, *P* < 0.001) was significantly higher in surgical patients, whereas the percentages of hematological malignancy (0.6% vs. 23.4%, *P* < 0.001) and DM (18.6%, vs. 37.5%, *P* = 0.002) were lower. As expected, TPN (94.2% vs. 40.6%, *P* < 0.001), the presence of a urinary catheter (73.8% vs. 32.8%, *P* < 0.001), and the presence of a gastric tube (76.7% vs. 35.9%, *P* < 0.001) were remarkably more frequent in surgical patients with candidemia versus non-surgical patients. Moreover, a higher rate of previous antibiotic therapy was documented for surgical patients than for controls (96.5% vs. 81.3%, *P* < 0.001). Instead, the proportion of renal replacement therapy in surgical patients was lower when compared to non-surgical patients (4.1% vs. 23.4%, *P* < 0.001). However, the proportion of patients who had ICU stay or received the presence of a CVC and mechanical ventilation, and 30-day mortality was similar between the two groups. Regarding laboratory findings, renal failure (18% vs. 40.6%, *P* < 0.001), leukopenia (11% vs. 29.7%, *P* = 0.001), and thrombocytopenia (14.0% vs. 37.5%, *P* < 0.001) were less common in surgical patients with candidemia than in controls.

### Antifungal susceptibility testing

Table 2 details antifungal susceptibility results of *Candida* spp. isolated from surgical patients with candidemia. Among the 172 isolates tested, overall resistance to fluconazole was 3.5%, susceptibility to fluconazole was 73.3%, and susceptible dose dependent (SDD) or intermediate susceptibility to fluconazole was 19.8%. For fluconazole, the highest susceptibility rate was identified in *C. parapsilosis* (87.8%), followed by *C. albicans* (86.2%) and *C. tropicalis* (78.6%). Isolates of *C. glabrata* exhibited the greatest proportion of SDD to fluconazole (100%), whereas the highest resistance rates were found in *C. krusei* (100%) and *C. tropicalis* (14.3%). Fluconazole susceptibilities of *Candida* isolates from candidemia with prior exposure to antifungal agents significantly decreased than those without (52% vs. 76.9%, *P* = 0.009). Overall, only 47.7% of isolates tested were susceptible to itraconazole. Furthermore, overall, 4.1% of isolates tested were resistant to voriconazole, and no resistance to amphotericin B was observed in our study.

**Table 2:** In vitro antifungal susceptibility of *Candida* species isolated from surgical patients with candidemia

Species	Antifungal agent	MIC (μg/ml)			No. (%) of isolates by new CBPs^**a**^		
**(n = 172)**		**Ranges**	**MIC**_**50**_	**MIC**_**90**_	**S**	**SDD / I**	**R**
*C. albicans* (*n* = 58)	Fluconazole	≤0.5–16	≤1	4	50 (86.2%)	6 (10.3%)	2 (3.4%)
	Voriconazole	≤0.03- ≤ 4	0.06	≤1	49 (84.5%)	3 (5.2%)	6 (10.3%)
	Itraconazole	0.062–1	≤0.125	0.25	NA	NA	NA
	Amphotericin B	≤0.25–1	≤0.5	0.5	58 (100%)	0	0
C. *parapsilosis* (*n* = 74)	Fluconazole	≤0.5–8	≤1	≤4	65 (87.8%)	8 (10.8%)	1 (1.4%)
	Voriconazole	≤0.03–0.25	≤0.06	0.125	72 (97.3%)	2 (2.7%)	0
	Itraconazole	≤0.062–0.25	≤0.125	0.125	72 (97.3%)	0	2 (2.7%)
	Amphotericin B	≤0.25–1	≤0.5	0.5	74 (100%)	0	0
C. *glabrata* (*n* = 19)	Fluconazole	≤1–16	4	16	0	19 (100%)	0
	Voriconazole	≤0.06–0.5	0.125	0.25	NA	NA	NA
	Itraconazole	≤0.125–1	0.25	1	NA	NA	NA
	Amphotericin B	≤0.25–1	≤0.5	0.5	19 (100%)	0	0
C. *tropicalis* (*n* = 14)	Fluconazole	≤1–32	1	16	11 (78.6%)	1 (7.1%)	2 (14.3%)
	Voriconazole	≤0.06–4	0.06	0.5	12 (85.7%)	1 (7.1%)	1 (7.1%)
	Itraconazole	≤0.125–8	0.125	1	10 (71.4%)	0	4 (28.6%)
	Amphotericin B	≤0.25–0.5	≤0.5	0.5	14 (100%)	0	0
*C. krusei*^b^ (n = 1)	Fluconazole	64			0	0	1 (100%)
	Voriconazole	0.5			1 (100%)	0	0
	Itraconazole	1			NA	NA	NA
	Amphotericin B	1			1 (100%)	0	0
Others (n = 6)	Fluconazole	≤1–16	≤2	16	NA	NA	NA
	Voriconazole	≤0.06–2	≤0.06	2	NA	NA	NA
	Itraconazole	≤0.125–1	0.125	1	NA	NA	NA
	Amphotericin B	≤0.5–0.5	≤0.5	0.5	NA	NA	NA

^a^ Specific clinical breakpoints (CBPs) for *Candida* susceptibility to fluconazole and voriconazole were determined following CLSI [14], while CBPs for susceptibility of *Candida* against itraconazole and amphotericin B were obtained from EUCAST [15]; ^b^ Minimum inhibitory concentration (MIC) 50 and MIC 90 values were not calculated for antifungal drugs against C. *krusei* owing to the small number of C. *krusei* case. Isolates of *C. krusei* are assumed to be intrinsically resistant to fluconazole. S, susceptible; I, intermediate; SDD, susceptible-dose dependent; R, resistant; NA, non-applicable;

### Antifungal therapy, outcomes and prognostic factors of candidemia in surgical patients

Of 172 episodes of candidemia occurred in surgical patients, empiric antifungal therapy was administered in 115 (66.9%) of the cases. The median duration of antifungal therapy was 9 (interquartile range 5–14) days in 145 surgical patients receiving any antifungal treatment. Among 27 (15.7%) surgical patients who did not receive any antifungal treatment, 5 patients died before or on the day the blood culture results were obtained. Among the remaining 22 patients without any antifungal treatment, the fever improved after surgical procedures, including CVC removal in 14 patients, the drainage catheter removal in four patients, left pyelostomy in two patients, the peritoneal drainage in one patient, and the endoscopic nasal biliary drainage in one patient. Fluconazole was the most commonly administered initial agent (88.3%), followed by micafungin (6.2%) and caspofungin (5.5%). The surgical patients with candidemia had lower 30-day mortality rates than those non-surgical patients (19.2% vs. 28.1%), although no statistic differences presented (*P* = 0.14). Regarding the 30-day mortality rates in the different surgeries, excepting the rate of 0% in patients undergoing urologic surgeries, gynecological and obstetric surgeries, the 30-day mortality rate was highest in patients undergoing thoracic surgeries (3, 50%) or undergoing neurosurgeries (2, 50%), followed by undergoing orthopedic surgeries (1, 33.3%), and undergoing general surgeries (27, 18.5%).

Within 30 days from the onset of candidemia, mortality occurred in 33 (19.2%) surgical patients, 2 of whom or their guardians decided to quit therapy after a few days of antifungal therapy, 5 of whom have died before the positivity of blood cultures was reported, 3 of whom died after the antifungal treatment, and 23 of whom died during the antifungal treatment. Univariable analyses identified several factors associated with 30-day mortality of surgical patients with candidemia, including ICU stay, recent abdominal surgery, recent cancer surgery, presence of CVC, renal failure, hypoalbuminemia, hyponatremia, thrombocytopenia, *C. albicans* infection and *C. parapsilosis* infection (Table 3). The results of the multivariable analysis for the risk factors associated with 30-day mortality are presented in Table 4. ICU stay (adjusted odds ratio (aOR) 6.55; 95% confidence interval (CI) 1.85–23.22; *P* = 0.004), thrombocytopenia (aOR 5.72; CI 1.54–21.20; *P* = 0.009), and *C. albicans* infection (aOR 6.08; CI 1.53–24.19; *P* = 0.01) were independent risk factors associated with increased 30-day mortality of surgical patients with candidemia.

**Table 3:** Univariable analysis of factors associated with 30-day mortality of surgical patients with candidemia

Variable	Survivors ***n*** = 139 (80.8%)	Deaths ***n*** = 33 (19.2%)	Odds ratio	95% Confidence interval	***P*** value
Age	61.6 ± 14.4	66.5 ± 12.6	1.03	1–1.06	0.07
Age ≥ 65 years	57 (41.0%)	18 (54.5%)	1.73	0.8–3.71	0.16
Male sex	97 (69.8%)	22 (66.7%)	0.87	0.39–1.95	0.73
Underlying diseases					
Solid tumors	77 (55.4%)	13 (39.4%)	0.52	0.24–1.14	0.10
Diabetes mellitus	24 (17.3%)	8 (24.2%)	1.53	0.62–3.81	0.36
Predisposing factors					
Current and former smokers	42 (30.2%)	10 (30.3%)	1	0.44–2.29	0.99
ICU stay	38 (27.3%)	25 (75.8%)	8.31	3.45–20.01	< 0.001
Recent abdominal surgery	118 (84.9%)	23 (69.7%)	0.41	0.17–0.98	0.046
Recent cancer surgery	74 (53.2%)	11 (33.3%)	0.44	0.2–0.97	0.04
Prior antibiotics exposure	134 (96.4%)	32 (97.0%)	1.19	0.13–10.58	0.87
Prior antifungal exposure	20 (14.4%)	5 (15.2%)	1.06	0.37–3.08	0.91
TPN	130 (93.5%)	32 (97.0%)	2.22	0.27–18.13	0.46
Presence of CVC	49 (35.3%)	18 (54.5%)	2.2	1.02–4.75	0.04
Mechanical ventilation	15 (10.8%)	5 (15.2%)	1.48	0.5–4.4	0.48
Presence of urinary catheter	106 (76.3%)	21 (63.6%)	0.54	0.24–1.22	0.14
Presence of gastric tube	109 (78.4%)	23 (69.7%)	0.63	0.27–1.47	0.29
Laboratory findings					
Renal failure	17 (12.2%)	14 (42.4%)	5.29	2.24–12.46	< 0.001
Anemia	116 (83.5%)	28 (84.8%)	1.11	0.39–3.18	0.85
Hypoalbuminemia	47 (33.8%)	24 (72.7%)	5.22	2.25–12.13	< 0.001
Hyponatremia	67 (48.2%)	6 (18.2%)	0.24	0.09–0.61	0.003
Leukopenia	14 (10.1%)	5 (15.2%)	1.59	0.53–4.79	0.41
Thrombocytopenia	10 (7.2%)	14 (42.4%)	9.51	3.7–24.42	< 0.001
*C. albicans* infection	39 (28.1%)	19 (57.6%)	3.48	1.59–7.62	0.002
*C. parapsilosis* infection	67 (48.2%)	7 (21.2%)	0.29	0.12–0.71	0.007
*C. glabrata* infection	15 (10.8%)	4 (12.1%)	1.14	0.35–3.69	0.83
*C. tropicalis* infection	11 (7.9%)	3 (9.1%)	1.16	0.31–4.43	0.82
Fluconazole susceptibility	103 (74.1%)	23 (69.7%)	0.8	0.35–1.85	0.61
Empirical antifungal therapy	94 (67.6%)	21 (63.6%)	0.84	0.38–1.85	0.66
No antifungal therapy	22 (15.8%)	5 (15.2%)	0.95	0.33–2.73	0.92
Outcome					
LOS (days)	33 (22–48)	31 (23–41.5)	1	0.98–1.01	0.52

ICU, Intensive care unit; TPN, Total parenteral nutrition; CVC, central venous catheter; LOS, Length of hospital stay;

**Table 4:** Multivariable logistic regression analysis of risk factors for 30-day mortality of surgical patients with candidemia

Variable	Adjusted odds ratio	95% Confidence interval	*P* Value
Age	1.04	1.0–1.08	0.06
Solid tumors	0.89	0.07–10.71	0.93
ICU stay	6.55	1.85–23.22	0.004
Recent abdominal surgery	0.33	0.09–1.24	0.1
Recent cancer surgery	0.67	0.05–8.88	0.76
Presence of CVC	0.98	0.28–3.38	0.97
Renal failure	1.78	0.54–5.95	0.35
Hypoalbuminemia	2.78	0.94–8.2	0.06
Hyponatremia	0.35	0.1–1.24	0.10
Thrombocytopenia	5.72	1.54–21.20	0.009
*C.albicans* infection	6.08	1.53–24.19	0.01
*C. parapsilosis* infection	1.07	0.24–4.7	0.93

CI, confidence interval; ICU, Intensive care unit; CVC, central venous catheter;

## Discussion

The present study focused on candidemia in surgical patients, and it showed that the proportion of surgical patients in the overall candidemia cases reached to 72.9%, which was much higher than around 50–55% reported in Europe, America, and Australia [4, 5, 9]. Moreover, our data also found the incidence of candidemia in surgical patients was 24.8 episodes per 1000 patient-days. This was dramatically higher compared with that observed in previous studies from North America and Europe, which has documented that the incidence density occurring in surgical ICU is nearly 0.6–0.98 episodes per 1000 patient-days [6, 16]. The variations in the incidence and the species distribution of candidemia may because the epidemiology of candidemia varied with geographic regions, type of hospital, patient populations, and study periods [8, 17].

Our study showed that the proportion of solid tumors was significantly higher in surgical patients than that in non-surgical patients. In solid-tumor patients with candidemia, several studies observed a predominance of *C. albicans* (around 41–53.7%) and the variable proportion of *C. parapsilosis*, *C. glabrata*, and *C. tropicalis*, which were 9.7, 20.2, and 7.1% in France [18], 20.7, 19.2, and 7.6% in Spain [19], 15, 12, 20% in Brazil [20], respectively. Nonetheless, we did not observe important differences regarding the distribution of *Candida* spp. between overall surgical patients and those with solid tumors. In the present study, the proportion of *C. glabrata* (11%) and *C. tropicalis* (8.1%) were relatively lower, whereas *C. parapsilosis* exceeded that *C. albicans* (33.7%) to account for 43% of all isolates among surgical patients. In contrast to our findings, *C. albicans* was the most frequently isolated spp. from candidemia, and *C. albicans* and *C. parapsilosis* were observed with a frequency of 51 and 25% among surgical patients in Italy [12], 50.9 and 21.8% in surgical wards in Spain [11], and 48 and 7% among surgical ICU patients in the United States [6], respectively. Of note, we observed the frequency of *C. parapsilosis* isolates among surgical patients was higher than those among non-surgical patients. *C. albicans* with 39.1% was the predominant species isolated in non-surgical patients in our study, followed by *C. parapsilosis* with 23.4%, *C. tropicalis* with 20.3%, and others. *C. parapsilosis* candidemia has been associated with CVC, TPN, prior antifungal therapy, neonates, and transplantation [17, 21–23]. Additionally, the transmission via the hands of health care workers, other medical devices and catheters may also be the risk factors for the nosocomial acquisition of *C. parapsilosis* infection [23]. The reasons for the high prevalence of candidemia due to *C. parapsilosis* among surgical patients are not completely understood, but the high use of invasive procedures and TPN in our study may partially explain the results. And thus, the optimal implementing device and catheter care may contribute to reduce episodes of *C. parapsilosis* BSI in our hospital. On the other hand, our findings showed the frequency of *C. tropicalis* candidemia was significantly lower in surgical patients than non-surgical patients. Moreover, *C. tropicalis* candidemia was found more frequently in surgical patients older than 65 years or with DM or in non-surgical patients with hematological malignancies, which is similar to that reported in previous studies [19, 20, 24, 25].

In our series, the proportion of fluconazole-susceptible *Candida* isolates in surgical patients did not significantly differ from that observed in non-surgical cases. Overall, on the basis of new species-specific CBPs, azole susceptibilities of *Candida* spp. were strikingly lower than the previously findings reported in the USA [26], parts of Italy and Spain [24], and Asian [27], but were similar to those observed in a prospective population-based surveillance for candidemia in Spain [4]. The elevated rates of fluconazole*-*SDD *C. albicans*, *C. parapsilosis*, and *C. glabrata* isolates emphasize the importance of the suitable and adequate systemic antifungal therapy for candidemia, as highly recommended in the guideline [28]. Our data confirmed that azole-resistant isolates were mainly observed in *C. tropicalis* and *C. krusei*, while amphotericin B had an excellent activity against all *Candid*a spp. tested. As expected, the *C. krusei* isolate, which is intrinsically resistant to fluconazole, was demonstrated to be susceptible to voriconazole. Although fluconazole prophylaxis has been reported to reduce the incidence of invasive candidiasis in high-risk patients in the surgical ICU [6, 29], previous exposure to fluconazole in high-risk surgical patients was also observed to be associated with subsequent candidemia caused by fluconazole-resistant *Candida* isolates [30], in agreement with our results. Despite the absence of antifungal susceptibility testing of echinocandins in current study, many studies have reported low resistance to echinocandins in *Candida* isolates, except for *C. parapsilosis* [4, 24, 26]. Due to the good safety profile, few drug-drug interactions and good susceptibilities, echinocandins have been recommended as first-line agents to treat invasive candidiasis [28, 31]. However, considering high treatment costs of echinocandins and amphotericin B, and more serious adverse effects of the latter, fluconazole was the most commonly prescribed antifungal agents for primary treatment in our study. Indeed, the use of amphotericin B is limited due to its severe side-effects in clinical practice, and thus echinocandins were commonly taken into consideration in the empirical antifungal treatment when candidemia may be caused by non-susceptible *Candida* isolates [28, 31]. After susceptibility results to antifungal drugs were reported, for fluconazole-susceptible isolates, if the patients were neither critical ill nor had the previous exposure to antifungal drugs, fluconazole was considered to be an alternative to an echinocandin.

The association between prior surgery, especially prior abdominal surgery and a better outcome in candidemia patients has been observed in other studies [10, 11, 32–34], which may be due to the lower severity of the underlying comorbidities, prompt antifungal therapy, and infection source control by CVC removal or appropriate surgical intervention [11, 33]. As mentioned previously, 30-day mortality of our surgical patients with candidemia were also lower than that of non-surgical patients. However, the difference did not reach statistical significance, probably because of similar percentages of ICU stay and *C. albicans* BSI between surgical and non-surgical patients, which were the risk factors for candidemia-related mortality in current study. The overall 30-day mortality in our surgical patients with candidemia was 19.2%, which is similar to that reported in surgical wards and ICU in northeastern Italy [33]. It was relatively high for candidemia patients with recent surgery (including ICU) in central Italy [12] and those in surgical ICU in France [10], which was 38 and 45.2%, respectively. In addition, the 30-day mortality of patients with candidemia hospitalized in surgical wards was observed to be 15.8% in a recent multicenter population study on candidemia in Spain [11], whereas it was as high as 38.7% in a retrospective study in Israel [35].

Not surprisingly, consistent with our data, ICU stay has been demonstrated to be independently associated with greater mortality in patients due to *Candida* BSI [12, 36], suggesting that severity of illness may affect the outcome of patients with candidemia. In agreement with previous reports [37, 38], we found that *C. albicans* was independently related to a poor outcome in surgical patients with candidemia, probably because of the relative virulence of *C. albicans*, patient’s underlying diseases and host defense deficiencies [39]. Among surgical patients with candidemia, our multivariable analysis also revealed that thrombocytopenia was independently associated with 30-day mortality, as have been described in adult patients with candidemia by Jia et al [36]. Except for their key role in thrombosis and hemostasis, platelets play a crucial role in inflammation, immunity, and the infectious disease [40]. Actually, the responses of platelets to bacterial infections are complicated, and thus thrombocytopenia can be either harmful or protective in host defense against pathogens depending on the pathophysiological context [41]. In any case, thrombocytopenia remained correlated to mortality of sepsis patients [40]. The platelet decrease in sepsis may be due to reduced thrombopoiesis, hemodilution, sequestration, the depletion of platelets, and immune-mediated destruction of platelets [40]. However, the mechanisms for platelet decrease and platelet function in candidemia have not been completely elucidated, and further studies are necessary.

There were several limitations to our study. First, due to the limitations of a retrospective cohort design, some variables cannot be analyzed. Second, echinocandin susceptibilities were not assessed since their minimum inhibitory concentration (MIC) CBPs were not interpreted by the CLSI or EUCAST at the time. And echinocandin regimens were empirically administered according to the CLSI or EUCAST document. Furthermore, the present study was performed at three hospital campuses and the results may not be applicable to other settings.

## Conclusions

In conclusion, our study showed that *C. parapsilosis* was the most common species surpassing *C. albicans*, suggesting the high prevalence of non-*C. albicans* species in surgical patients with candidemia. Moreover, among patients with recent surgery, risk factors for candidemia caused by various *Candida* species and resistance of these isolates to azoles, particularly fluconazole, should be strongly considered when starting an empirical treatment. The recognition of prognostic factors for candidemia in surgical patients may help clinicians to assess and prevent poor outcomes.

## Data Availability

The datasets used in this study are available from the corresponding author upon reasonable request.

## Abbreviations

BSI: Bloodstream infection
CVC: Central venous catheter
ICU: Intensive care unit
CBP: Clinical breakpoints
CLSI: Clinical Laboratory Standards Institute
EUCAST: European Committee on Antimicrobial Susceptibility Testing
MIC: Minimum inhibitory concentration
OR: Odds ratios
CI: Confidence intervals
DM: Diabetes mellitus
LOS: Length of hospital stay
TPN: Total parenteral nutrition
SDD: Susceptible dose dependent
